# Childhood maltreatment and self-harm in Chinese adolescents: moderation and mediation via resilience

**DOI:** 10.1186/s12889-021-11605-y

**Published:** 2021-08-17

**Authors:** Xin Tian, Jin Lu, Yusan Che, Die Fang, Hailiang Ran, Xingting He, Yanjiao Wang, Tianlan Wang, Xiufeng Xu, Guangya Yang, Yuanyuan Xiao

**Affiliations:** 1 Department of Epidemiology and Health Statistics, School of Public Health, Kunming Medical University, Kunming, 650500 Yunnan China; 2 The First Affiliated Hospital of Kunming Medical University, Kunming, Yunnan China; 3 NHC Key Laboratory of Drug Addiction Medicine, The First Affiliated Hospital of Kunming Medical University, Kunming, China; 4 Lincang Psychiatry Hospital, Lincang, Yunnan China

**Keywords:** Childhood maltreatment, Self-harm behaviors, Resilience, Emotion regulation, Family support, Interpersonal assistance

## Abstract

**Background:**

Published studies examining the association between childhood maltreatment (CM) and self-harm (SH) among adolescents have been accumulated. It is possible that resilience serves as a moderator or mediator in CM-SH association, nevertheless, this topic has never been thoroughly investigated.

**Methods:**

In this population-based cross-sectional study, we surveyed 3146 students aged 10–17 in southwest China. The Childhood Trauma Questionnaire (CTQ), the Modified version of Adolescents Self-Harm Scale (MASHS), and the Resilience Scale for Chinese Adolescents (RSCA) were used to measure CM, SH, and resilience. Correlational analyses, hierarchical multivariate linear regression, and structural equation modeling (SEM) were performed to test the moderation and mediation of resilience in CM-SH association.

**Results:**

Findings revealed that, resilience with its five dimensions, CM, and SH were significantly correlated with each other. Resilience partially moderated and mediated the association between CM and SH. Besides, among all dimensions of resilience, emotion regulation, interpersonal assistance, and family support presented the strongest mediation in CM-SH association.

**Conclusions:**

Our results highlight the importance of resilience in CM related SH among Chinese teenagers. Resilience-oriented intervention could be considered in SH intervention measures for adolescents who had experienced CM.

## Background

Childhood maltreatment (CM) is physical, sexual, psychological abuse or neglect of children, especially by parent(s) or caregiver(s). CM has been identified as a major public health issue. It is reported that each year, 4–16% and 5–10% of children are physically and sexually abused, and one-tenth of children suffered from emotional abuse or neglect by their parents [1]. CM is also prevalent in China: a single site survey of 7702 participants revealed that, for children under 16 years old, a staggering 31.1% reported had been abused [2]. CM can impose multiple detrimental impacts on the victims, from deficits in educational achievement, mental problems (such as depression, anxiety, post-traumatic stress symptoms), to low self-esteem, dissociation, and personality disorders, all of which can persist into the adulthood [1, 3].

Self-harm (SH) normally defined as intentional, direct destruction of body tissue, with or without suicidal intent [4]. Although it can occur within any period of life, a higher prevalence has been found in pubertal age [5]. SH has long been considered a major problem among youths [6], affecting 17% of those aged between 12 and 18 years in China [7]. Though non-fatal, SH always indicative of tragic outcomes: it has been repeatedly verified as the strongest indicator of future suicide [8]. Besides, following the index episode of SH, self-injurers are inclined to develop repeated and frequent SH, and about 2–7% of them will commit suicide in the following years [9, 10].

Among all adverse psychosocial factors, CM has been linked to elevated risk of SH in adolescents [11]. Given the fact that, CM can increase not only the risk of SH but also the probability of suicidal behaviors [12], CM related SH in youths should be effectively intervened. Except for direct measures on preventing CM in general, interventions which targeting on crucial mediators or moderators in CM-SH association can also be considered. Resilience is gradually attracting attention in the recent years, especially among children and adolescents. It reflects the ability to well adapt the adversities [13]. It has been found that resilient individuals tended to report decreased risk in negative consequences (like depression, anxiety) after exposed to CM [14]. Similarly, a higher level of resilience was related to lower risk of internalizing problems [15]. Therefore, it is reasonable to postulate that resilience may also protect against SH among adolescents who had experienced CM [16]. Also, a meta-analysis concluded that, the detrimental effect of CM can be moderated or even averted under the premise of effectively consolidated resilience of the victims [14]. Therefore, we put forward the assumption that, resilience plays a prominent role in CM-SH association, possibly as a mediator, or a moderator, or both. Nevertheless, no published studies have investigated this assumption so far.

In this cross-sectional study, we intended to discuss this issue in a large representative sample of Chinese adolescents. We tested the hypothesized mediation and moderation of resilience in the association between CM and SH.

## Method

### Participants

Participants in this cross-sectional study were recruited from 14 schools (5 primary schools, 5 junior middle schools, and 4 high schools) in Lincang, Yunnan, China, from December 1 to 13, 2019. A three-stage random clustering sampling method was applied in the present survey: In stage one, we randomly chose Linxiang district out from 8 prefectures and districts within Lincang’s jurisdiction; in stage two, 14 schools in Linxiang district were randomly determined; in stage three, all eligible students (aged 10–17) from 3 to 4 randomly selected classes within each chosen school were initially included.

### Measurements

Each participant was invited to fill a self-administered questionnaire that contains demographics, CM, resilience, SH, bullying, negative life events, etc. Only the former four parts were analyzed in the present study. Prior to the survey, informed consents from the participants and their legal guardians were obtained. This study was approved by the Ethics Committee of The Third People’s Hospital of Lincang (Lincang Psychiatry Hospital).

#### Childhood maltreatment

The brief version of Childhood Trauma Questionnaire (CTQ) is comprised of 28 questions [17], and can be divided into five subscales, measuring three types of abuse (physical, sexual, emotional) and two types of neglect (emotional and physical). A 5-point Likert scale was used to gauge the frequency of maltreatment. The Cronbach’s ɑ for CTQ in the present study was 0.81 (Bootstrap 95% CI: 0.80–0.83). The authors had received permission to use the questionnaire.

#### Resilience

The Resilience Scale for Chinese Adolescents (RSCA) is a 28-item instrument [18], assessing five dimensions of resilience (goal concentration, interpersonal assistance, emotion regulation, family support, and positive perception). Answers to each question are 5-point Likert style, from “completely disagree” (with assigned score of 1) to “completely agree” (with assigned score of 5). A higher combined score of RSCA as well as its five dimensions indicate a better resilience in general or in specific aspect. The Cronbach’s ɑ of the RSCA in the present study was 0.86 (Bootstrap 95% CI: 0.85–0.87). The authors had received permission to use the questionnaire.

#### SH

The Modified version of Adolescents Self-Harm Scale (MASHS) simultaneously evaluating the frequency and severity of the 18 most commonly seen SH behaviors in Chinese adolescents [19]. Response categories for frequency and severity were from “never” (with assigned score of 1) to “higher than five times” (with assigned score of 4) and from “non-perceptible injury” (with assigned score of 1) to “extremely critical injury” (with assigned score of 5). The authors had received permission to use the questionnaire.

### Data analytic procedure

The demographic and psychometric variables were summarized using descriptive statistics. A Pearson correlation matrix between CM (CTQ), resilience with its five dimensions (RSCA), and SH score (MASHS) was then conducted to examine pairwise correlations. After centering scores of CTQ, RSCA, and MASHS, a series of hierarchical regression models which controlled for significant demographics identified in univariate analysis were fitted: SH score was taken as the dependent variable, the adjusted association between CM and SH, resilience and SH were estimated, besides, the product term of CM and resilience was finally incorporated to explore the moderating effect of resilience in CM-SH association. Finally, two path models were established to test for the mediation of resilience as well as its five dimensions.

All analyses were executed by using the R software (Version 3.6.2, The R Foundation for Statistical Computing, Vienna, Austria). The “survey” and “lavaan.survey” packages were used to recalibrate the unequal sampling weights produced by cluster sampling design.

## Results

### Descriptive statistics

After excluding ineligible participants (failed quality inspection: *N* = 7; failed age requirement: *N* = 88), a total of 3146 were entered into our analyses with an effective response rate of 97.07%. The basic characteristics of the respondents were provided in Table 1: 1480 adolescents endorsed at least one episode of SH, and the lifetime prevalence was 47.07% (95% CI: 45.33–48.81%). The mean scores for MASHS and CTQ were 10.18 (SD = 20.59) and 36.03 (SD = 9.59), respectively. Regarding to resilience, the average scores of the five dimensions varied from 13.58 (SD = 4.13) to 22.42 (SD = 4.71), while the average RSCA score was 95.52 (SD = 17.73).

**Table 1:** Demographic characteristics of all study samples (*N* = 3146)

Factor	*N* (%)	Mean ± *SD*
**Sex**		
Male	1437 (45.68)	
Female	1709 (54.32)	
**Age**		13.32 ± 2.18
**Ethinicity**		
Han	2113 (67.16)	
Yi	365 (11.60)	
Wa	103 (3.27)	
Others	565 (17.96)	
**Grade**		
Primary school	1132 (35.98)	
Middle school	1069 (33.98)	
High school	945 (31.04)	
**Age of father**		42.27 ± 5.02
**Age of mother**		39.49 ± 4.73
**Educational level of father**		
Primary school and below	885 (28.13)	
Middle school	1006 (32.01)	
High school or equivalent and above	926 (29.44)	
Unknown	245 (7.79)	
Missing	84 (2.67)	
**Educational level of mother**		
Primary school and below	1077 (34.23)	
Middle school	951 (30.23)	
High school or equivalent and above	865 (27.50%)	
Unknown	223 (7.09)	
Missing	30 (0.95)	
**CTQ**	36.03 (9.59)	
**RSCA total**	95.52 (17.73)	
Goal concentration		17.21 ± 5.09
Interpersonal assistance		21.18 ± 5.67
Emotion regulation		21.14 ± 5.65
Positive perception		13.58 ± 4.13
Family support		22.42 ± 4.71
**Self-harm behavior (Yes)**	1480 (47.04%)	
**Self-harm level score**	10.18 (20.59)	

### Correlations between childhood maltreatment, resilience, and SH

**Fig. 1:**
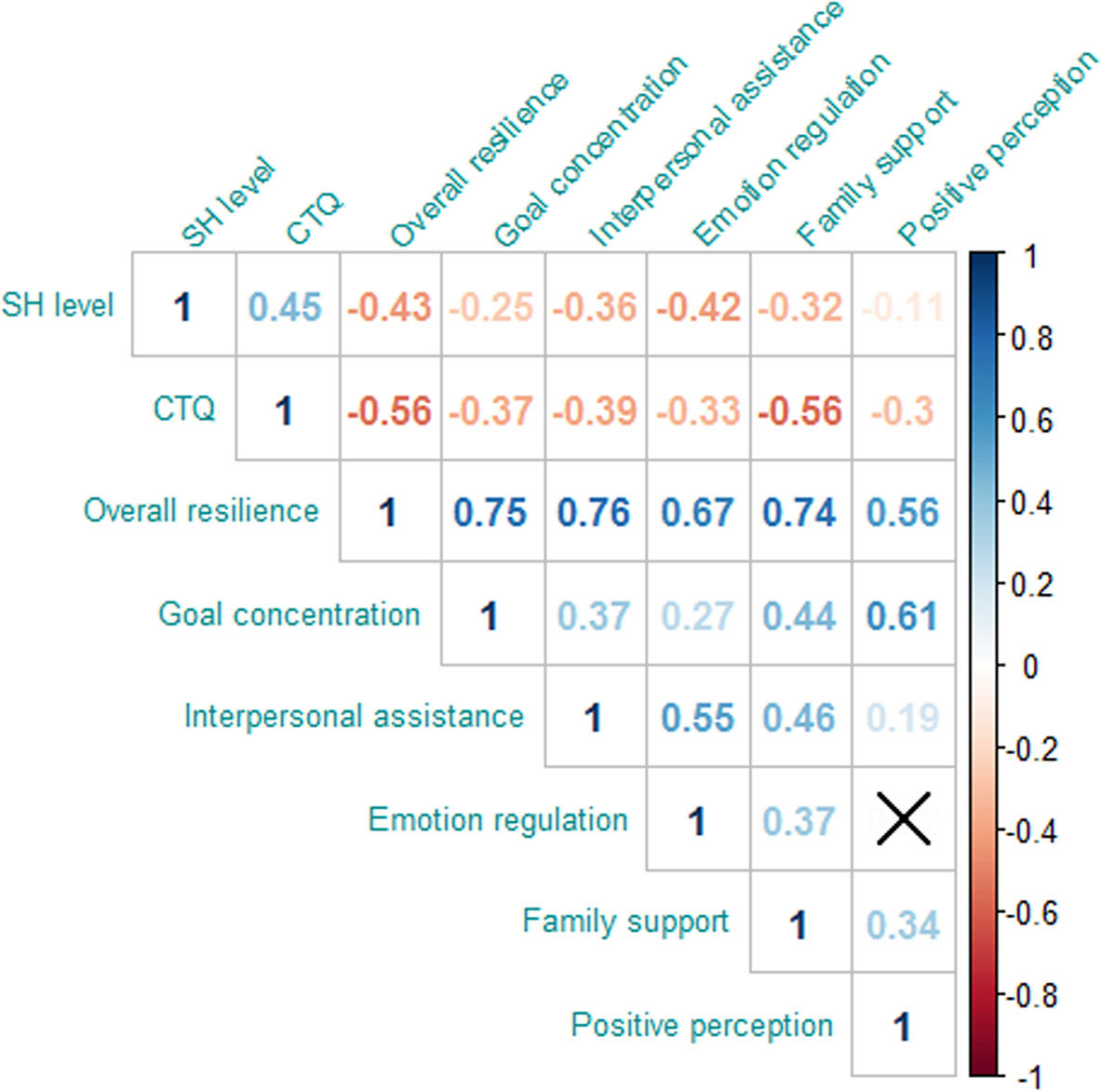
Correlation matrix for childhood maltreatment (CTQ), resilience with five dimensions and SH levels. Note: SH, self-harm; CTQ, The childhood trauma questionnaire. All presenting correlation coefficients’ *p* values< 0.001; ‘×’: *p* > 0.05

As illustrated in the correlation matrix (Fig. 1), the scores for CTQ, RSCA, and MASHS were significantly correlated with each other: resilience in general, together with all its 5 dimensions, were significantly inversely correlated with both CM and SH, with correlation coefficients ranging from − 0.11 (*p* < 0.001) to − 0.56 (*p* < 0.001); a significant positive correlation was presented between CTQ score and MASHS score (*r* = 0.45, *p* < 0.001).

### Moderation of resilience in CM-SH association

**Fig. 2:**
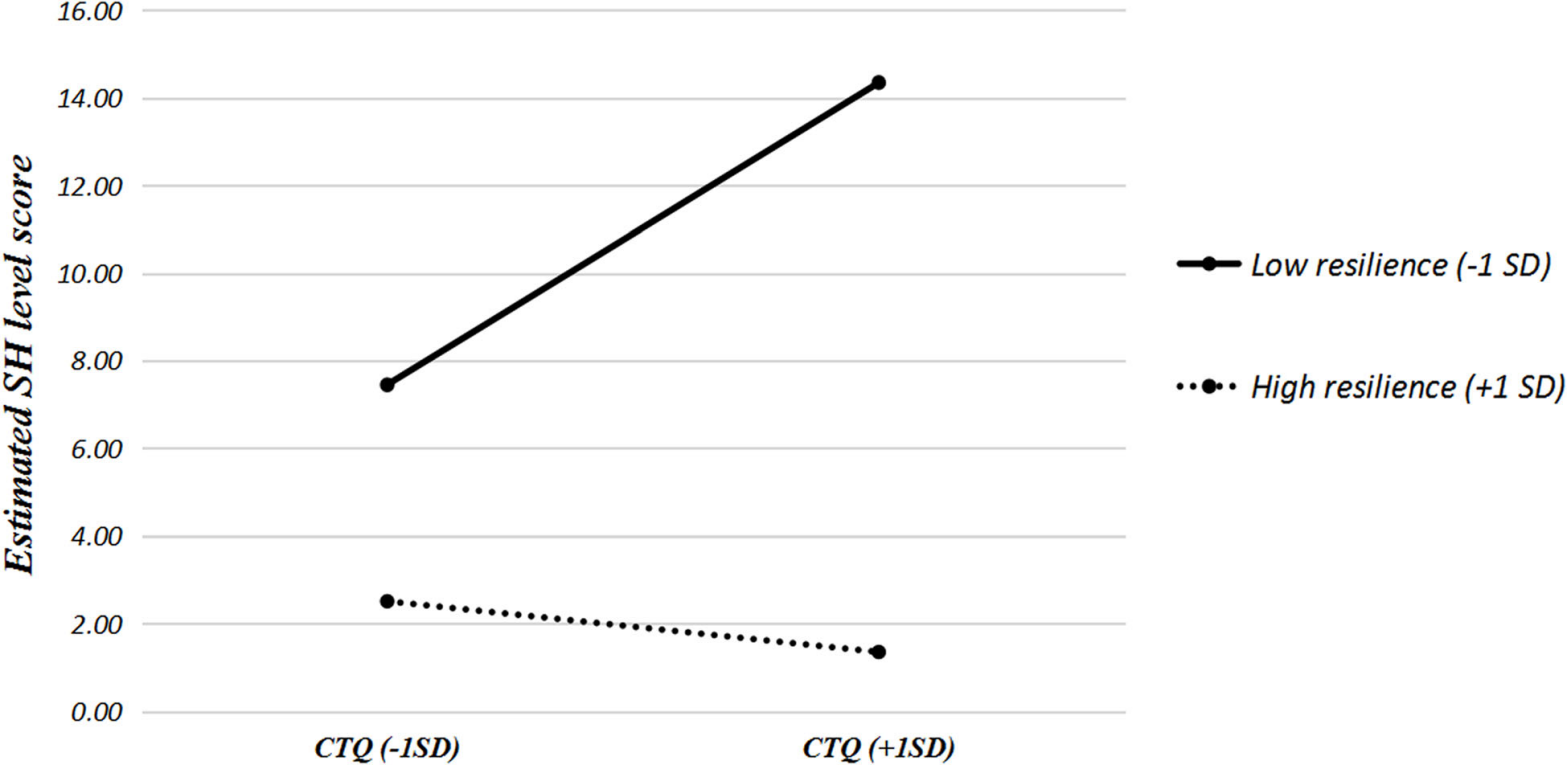
The estimated SH level scores for adolescents who scored low (− 1 SD) vs. high (+ 1 SD) on CTQ and low (− 1 SD) vs. high (+ 1 SD) on RSCA. Note: SH, self-harm; CTQ, childhood trauma questionnaire, RSCA, The resilience scale for Chinese adolescents

The results of hierarchical multivariate linear regression model were displayed in Table 2. Scores for CTQ, RSCA, MASHS were centered to weaken multicollinearity. CTQ and demographic variables (sex, age, grade, educational levels of mother) were entered in the first model; resilience was then added in in the second model; in the third model, the interaction term of CM and resilience was incorporated. The results indicated that, both CM and resilience were significantly associated with SH. Moreover, it was found that the interaction between resilience and CM had a weak but significant association with SH, which indicated that resilience served as a moderator in CM-SH association: adolescents with lower resilience were found elevated SH scores under severer exposure of CM, whereas for adolescents of higher resilience, individuals with severer CM had decreased SH scores (Fig. 2).

**Table 2:** Hierarchical regression analyses for testing the moderation role of resilience

Covariates	Univariable	*p value*	Model 1	Model 2	Model 3
	B (95% CI)		B (95% CI)	B (95% CI)	B (95% CI)
Sex (Ref: male)					
Female	3.64 (1.29–5.98)	0.014	4.28 (2.34–6.22)	3.63 (1.96–5.24)	2.88 (1.42–4.34)
Age (+ 1)	1.61 (0.82–2.39)	0.003	0.24 (−0.68–1.16)	0.03 (−0.88–0.94)	0.01 (−0.07–0.80)
Ethnicity (Ref: Other)					
Han	− 0.39 (−2.44–1.66)	0.716	–	–	–
Grade (Ref: primary school)					
Middle school	6.88 (2.89–10.87)	0.009	6.84 (2.90–10.79)	6.89 (3.53–10.25)	6.50 (3.12–9.88)
High school	8.68 (3.72–13.64)	< 0.001	8.80 (1.26–16.34)	8.80 (1.84–15.76)	8.19 (1.80–14.58)
Age of father(+ 1 year)	0.17 (−0.04–0.38)	0.141	–	–	–
Age of mother(+ 1 year)	0.15 (−0.04–0.35)	0.163	–	–	–
Educational level of father (Ref: primary school and below)					
Middle school	0.21 (−1.32–1.75)	0.793	–	–	–
High school or equivalent and above	−1.67 (−4.72–1.37)	0.313	–	–	–
Educational level of mother (Ref: primary school and below)					
Middle school	−2.13 (−4.00–0.25)	0.016	0.34 (−1.52–2.20)	0.61 (− 1.04–2.26)	0.43 (− 1.33–2.18)
High school or equivalent and above	−3.04 (−6.24–0.16)	0.099	2.18 (0.11–4.24)	2.96 (1.13–4.80)	2.30 (0.43–4.18)
CTQ			1.02 (0.77–1.26)	0.71 (0.55–0.86)	0.42 (0.30–0.55)
RSCA (+ 5)			–	−1.47 (− 1.93- -1.01)	−1.67 (−2.11- -1.23)
CTQ*RSCA (+ 5)			–	–	−0.10 (−0.13- -0.07)

Note: CTQ, The childhood trauma questionnaire; RSCA, The resilience scale for Chinese adolescents

### Mediation of resilience in CM-SH association

**Fig. 3:**
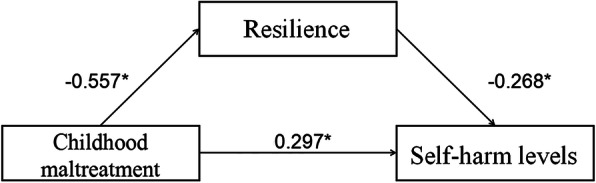
SEM analysis of the indirect effect of childhood maltreatment on self-harm levels via resilience. Note: All path coefficients were standardized. ^*^p < 0 .01

The preceding results were indicative of a potential mediation by resilience in CM-SH association, therefore we conducted structural equation modeling (SEM) to test this hypothesis. The hypothesized path model exhibited satisfactory fit to our data. As graphically presented in Fig. 3, CM was directly associated with resilience (*b* = − 0.56, *p* < 0.001) and SH level (*b* = 0.30, *p* < 0.001), resilience was directly associated with SH level (*b* = − 0.27, *p* < 0.001) and partially mediated the association between CM and SH (standardized indirect association = − 0.56*-0.27 = 0.15), which explained 33.46% of the total association (total association = − 0.56*-0.27 + 0.30 = 0.45).

**Fig. 4:**
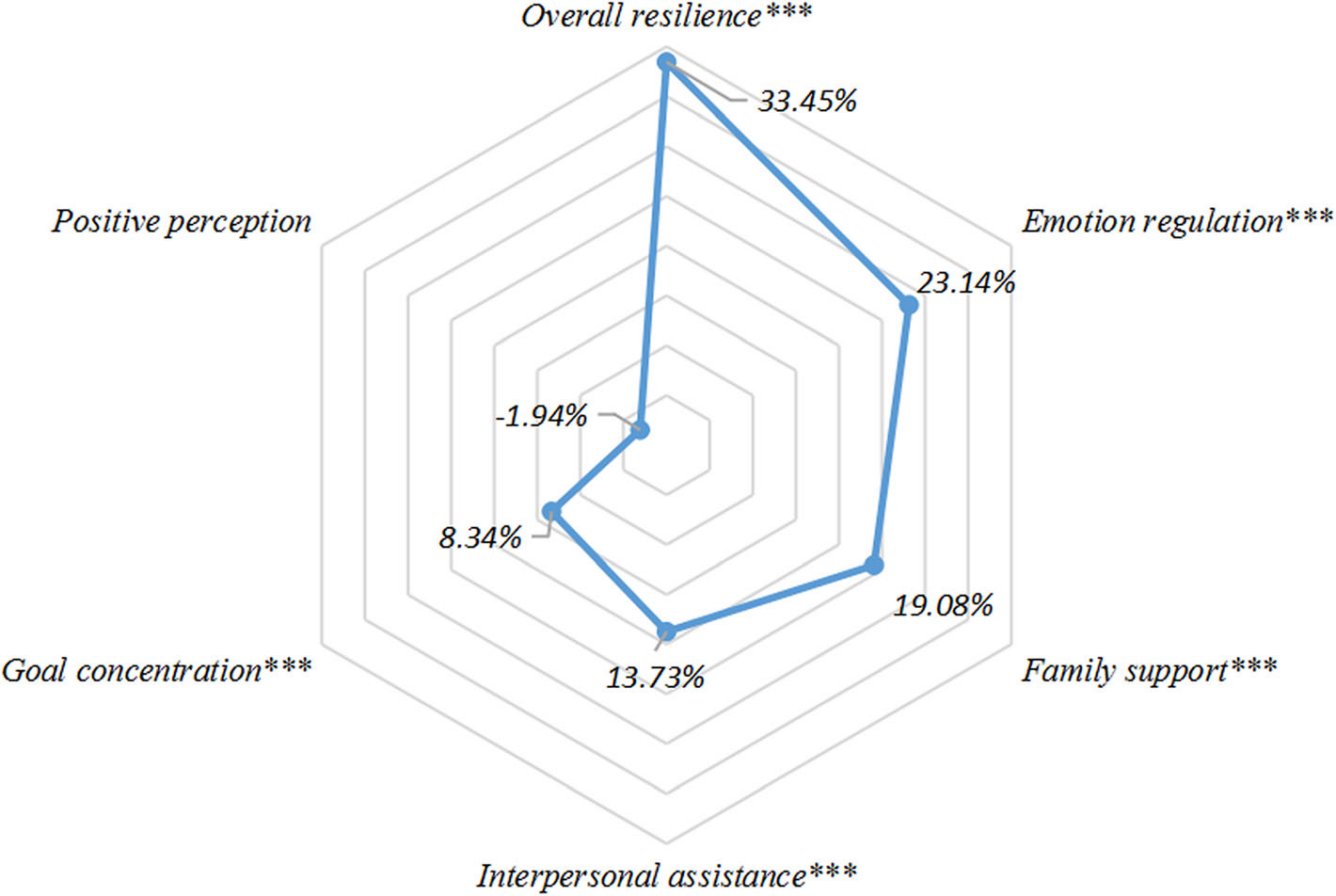
The mediating effect percentages of resilience with five dimensions on the relationship between childhood maltreatment and self-harm levels. ^***^p < 0.001

Due to the identified significant mediation via the overall resilience, a group of additional mediation analyses were conducted for the five dimensions of resilience (goal concentration, interpersonal assistance, emotion regulation, family support, and positive perception), and the results were collectively summarized in Fig. 4: except for positive perception, which presented an insignificant association with SH, all the other four dimensions were significant mediators in the association of CM-SH. Emotion regulation presented the most conspicuous mediation (23.14%), followed by family support (19.08%).

## Discussion

In agreement with our anticipation, resilience may simultaneously mediate and moderate the association between childhood maltreatment and self-harm in Chinese adolescents: although the moderation was week, nearly one-third of the CM-SH association can be mediated via resilience. Besides, except for positive perception, all the rest 4 dimensions of resilience presented as prominent mediators, specifically, emotion regulation and family support showed the strongest mediation.

As hypothesized, resilience was identified as a prominent moderator in CM-SH association, although the moderation was weak, to be specific, a higher resilience of the victim tended to attenuate this association. Existing literature is supportive of the interaction between resilience and childhood trauma in predicting depression [20–22], the most significant risk factor for SH [6]. According to the theory proposed by Nock, SH emerges with the attempt to cope with adverse events [16]. As a defensive mechanism to cope with adversity, resilience is built on the integration of physical, psychological, social, and intellectual resources [23]. These resources consequently are helpful for an individual to cope with environmental stresses [24], like childhood maltreatment. Therefore, it is possible that highly resilient adolescents are more capable of acquiring and mobilizing these resources to help them antagonizing the negative influence of abusive or neglectful family environments. In previous studies, researchers also found that more resilient victims of negative life encounters reported less severe psychological problems or SH engagement [25–27]. Working with a sample of boarding undergraduate students, however, Madden and Shannon did not find resilience as a moderating variable in the association between CM and SH [28]. One possible explanation could be that the limited sample size (*N* = 285) hampers statistical efficiency in detecting significant relationship. Moreover, drawn from a residential school, participants in that research enjoyed more chances to seek assistance or consult from their roommates in coping with CM, which may potentially reduce SH risk, as social support is considered a protective factor of SH among adolescents [29]. Future studies of larger sample sizes and longitudinal design are expected to further discuss this issue.

Another important finding is that, aside from moderation, resilience also presented as a strong mediator in CM-SH association. Moreover, specific dimensions of resilience acted discordantly in this mediation, with emotion regulation emerged as the strongest mediator. Attachment theory suggests that growing up in attachment threatening environments, such as abusive or neglectful families, children can exhibit a propensity to suppress their feelings instead of confiding to their parents. The long-term suppression in mood may impose a detrimental effect on emotion regulatory function [30, 31]. Besides, given the fact that self-injurers always adopt SH as a method to vent negative feelings [32], it is reasonable that emotionally dysregulated young victims of family abuse or neglect may resort to SH. Besides, individuals with childhood traumatic experiences presented increased risk in developing disordered sensory processing patterns, such as low self-registration, sensory sensitivity, and sensation avoidance, all are related to increased risk of depression [33–36], a well-recognized and most important risk factor of SH.

For other dimensions of resilience, family support and interpersonal assistance also showed conspicuous mediation in CM-SH association. From an empirical perspective, it is a consensus that children experienced CM are more difficult in establishing secure parent-child attachments [37]. Insecure parent-child relationship can lead to lower levels of self-esteem, self-efficacy, and reflective functioning [38–40], all of which increase vulnerability to mental symptoms like depression in adolescence [41–43]. Moreover, school-aged children with intimate parent-child bonds tend to have lower presence, frequency, and severity of SH [44]. Prospective studies have identified a connection between childhood abuse and future dysfunctional social relationships and interpersonal problems [45]. Therefore, it is possible that victims of CM may be less willing to seek assistance from the others under stressful situations, which may in turn result in an increased risk of SH.

Our results shed new light on intervention strategies of SH in adolescents with CM experiences. On one hand, measures aiming at reinforcing emotion regulation ability among maltreated youths may be effective in curtailing SH. Two types of intervention that focusing on improving emotion dysregulation to antagonize SH have been developed in clinical practice, one is dialectical behavior therapy, the other is acceptance-based emotion regulation group therapy. Besides, a telephone-based therapy has been found effective in positive emotion regulation among patients with bipolar depression [46, 47]. On the other hand, as both family support and interpersonal assistance are primary sources of social support, enhancing social support might be another promising direction in preventing CM associated SH among teenagers. At first, problem-solving therapy from which individuals can learn how to seek interpersonal assistance might be beneficial, as such therapy has been proved effective in reducing helplessness and suicidal ideation in depressive patients, though in small-sized samples [48]. However, evidence from a large pragmatic trial recommended problem-solving therapy as an alternative only for patients with repeated SH attempt [49]. Moreover, promoting positive comprehension of family support can also be considered, as an interventional study has proved its effectiveness among Chinese adolescents from impoverished areas [50].

Our study is among the first to test the moderating and mediating effect of resilience in the association between CM and SH in a large sample of Chinese adolescents. However, several limitations should be noticed. First, the cross-sectional nature prevents causal conclusions. Second, self-reported questionnaires are prone to information bias. Third, study subjects were chosen from a single province in southwest China, therefore a limit should be set for the generalization of study results. Future studies with expanded sampling sources and prospective design are warranted.

## Conclusion

In this cross-sectional study of Chinese adolescents, we found that childhood maltreatment was prominently associated with SH, besides, resilience exhibited significant moderation and mediation in CM-SH association. Among all dimensions of resilience, emotion regulation, family support and interpersonal assistance emerged as the strongest mediators. The present study offers new perspectives for the prevention of CM related SH among Chinese adolescents: measures for strengthening psychological resilience, especially for improving emotion regulation ability, consolidating family support and interpersonal assistance should be considered in reducing the risk of SH among adolescent CM victims.

## Data Availability

The datasets used and/or analyzed during the current study are available from the corresponding author on reasonable request.

## Abbreviations

CM: Childhood maltreatment
SH: Self-harm
CTQ: Childhood Trauma Questionnaire
RSCA: The Resilience Scale for Chinese Adolescents
MASHS: The Modified version of Adolescents Self-Harm Scale
